# Nomograms Predicting Survival of Cervical Cancer Patients Treated With Concurrent Chemoradiotherapy Based on the 2018 FIGO Staging System

**DOI:** 10.3389/fonc.2022.870670

**Published:** 2022-05-11

**Authors:** Qingyu Meng, Weiping Wang, Xiaoliang Liu, Dunhuang Wang, Fuquan Zhang

**Affiliations:** Department of Radiation Oncology, Peking Union Medical College Hospital, Chinese Academy of Medical Science and Peking Union Medical College, Beijing, China

**Keywords:** cervical cancer, radiation therapy, nomogram, FIGO stage, survival

## Abstract

**Background:**

In 2018, a revised staging system was released for cervical cancer, which defined pelvic and paraaortic lymph node metastasis as stages IIIC1 and IIIC2, respectively. In this study, we constructed and validated nomograms to predict the 3- and 5-year survival of patients with cervical cancer based on the revised International Federation of Gynecology and Obstetrics (FIGO) staging system.

**Methods:**

We retrospectively examined patients with 2009 FIGO stage IB–IVA cervical cancer who were treated at our institute between 2011 and 2015. Patients were randomized into the model development and validation cohorts (2:1). Univariate and multivariate analyses were conducted for the model development cohort to identify prognostic factors. In the multivariate analysis, nomograms were built to predict overall survival (OS) and disease-free survival (DFS) using significant variables. The nomograms were assessed based on the discrimination and calibration in both cohorts. Discrimination was assessed using the concordance index. Calibration was performed by comparing the mean nomogram estimated survival and the mean observed survival.

**Results:**

We included 1,192 patients, with 795 and 397 patients in the model development and validation cohorts, respectively. In the model development cohort, the median follow-up period was 49.2 months. After multivariate analysis, age, histology, 2018 FIGO stage, and pelvic lymph node number were independent factors for OS. Histology, 2018 FIGO stage, squamous cell carcinoma antigen, and pelvic lymph node number were significant predictors of DFS. The nomograms constructed to predict OS and DFS were based on these factors. In both model cohorts, the concordance index for the nomogram-predicted OS and DFS was 0.78 and 0.75 and 0.74 and 0.67, respectively. The calibration curve revealed good agreement between the nomogram predictions and actual values.

**Conclusion:**

We constructed robust nomograms to predict the OS and DFS of patients with cervical cancer undergoing treatment with concurrent chemoradiotherapy based on the 2018 FIGO staging system.

## Introduction

Cervical cancer is the fourth most frequently diagnosed cancer and fourth leading cause of cancer-related death among women worldwide (1). Lymph node metastasis (LNM) plays an important role in the metastasis of cervical cancer, and patients with cervical cancer with pelvic and paraaortic lymph node metastases have the worst survival rate (2). The International Federation of Gynecology and Obstetrics (FIGO) staging system is widely used in cervical cancer. Considering the limited imaging resources in developing countries, LNM was not included in the FIGO staging system before 2018 (3, 4). In 2018, FIGO revised its staging system for cervical cancer and defined pelvic LNM and paraaortic LNM as IIIC1 and IIIC2, respectively (5).

In the era of individualized cancer treatment, accurately estimating a cancer patient’s survival aids in the clinical decision-making process throughout their treatment. Compared with staging, nomograms can involve multiple prognostic factors, including staging, and estimate patient survival more accurately. Many nomograms have been constructed to predict the survival of patients with cervical cancer treated with concurrent chemoradiotherapy (6–11). However, most are based on the 2009 or 2014 FIGO staging system (3, 4). Therefore, they are not applicable per the updated 2018 FIGO staging system (5).

In this study, we evaluated patients with cervical cancer being treated at our institute with definitive radiotherapy or concurrent chemoradiotherapy and constructed nomograms predicting their overall survival (OS) and disease-free survival (DFS) based on staging using the 2018 FIGO system and additional factors.

## Methods

### Patients

The Institutional Review Board of Peking Union Medical College Hospital approved this study. We retrospectively examined cervical cancer patients treated with definitive radiotherapy or concurrent chemoradiotherapy between January 2011 and December 2015. The inclusion criteria were age ≥18 years, histologically proven cervical cancer (including squamous cell carcinoma (SCC), adenocarcinoma, and adenosquamous cell carcinoma), 2018 FIGO stage IB–IVA, and treated with concurrent chemoradiotherapy or definitive radiotherapy. Patients with previous surgery or receiving palliative radiotherapy were excluded.

### Treatment and Follow-Up

All patients were scheduled to receive external beam radiation therapy and intracavitary brachytherapy. The clinical target volume (CTV) covered the gross tumor, uterus, cervix, parametrium, upper part of the vagina, and pelvic lymph node region. For patients with paraaortic lymph node involvement or at high risk of its failure, this region was also covered in the CTV. The gross tumor volume included the involved lymph nodes. The planning clinical target volume was defined as the CTV plus a 6–10-mm margin. A margin of 5 mm was added to the gross tumor volume to form the planning gross tumor volume. Doses of 50.4 and 59–61 Gy were administered in 28 fractions to the planning CTV and planning gross tumor volume, respectively. High dosage intracavitary brachytherapy was performed using ^192^Ir. A 30–36-Gy dose administered in five to seven fractions was prescribed to point A. The first line of concurrent chemotherapy was a weekly regimen of cisplatin. The treatment protocol has been described previously (2, 12).

Follow-up examinations were performed every 3 months in the first 2 years, then every 6 months between 3 and 5 years, and once a year after 5 years.

### Statistics

To develop and validate nomograms to predict OS and DFS, patients were randomized into model development and validation cohorts (2:1). Their basic characteristics were compared using the Chi-square test, continuity correction, or Fisher’s exact test, as appropriate.

To identify potential predictors of OS and DFS, the following characteristics were included in the univariate analysis: age (grouped into young <65 years and elderly ≥65 years), histology (grouped into SCC and non-SCC), tumor size (grouped into <4 and ≥4 cm), the 2018 FIGO stage (grouped into IB, IIA, IIB, IIIA, IIIB, IIIC1, IIIC2, and IVA), SCC antigen (grouped into <8.6 and ≥8.6 ng/ml), pelvic lymph node number (grouped into 0–1 and ≥2), and common iliac LNM (grouped into yes and no). FIGO stage was recorded using the 2009 or 2014 FIGO staging system and was subsequently transposed into the 2018 FIGO staging system.

SCC antigen was grouped into <8.6 and ≥8.6 ng/ml based on a previous study (13). Lymph node metastasis was diagnosed using standard imaging approaches. Lymph nodes with short diameters (≥1 cm) or those confirmed as having tumors on positron emission tomography/computed tomography were defined as metastatic lymph nodes.

The Kaplan–Meier method was used to estimate OS and DFS. Univariate and multivariate analyses were performed using a Cox regression model. Significant factors in the univariate analysis were incorporated into the multivariate analysis. Multivariate analysis was performed using the backward method. Nomograms to predict OS and DFS were built using the significant variables from the multivariate analysis. The nomograms were assessed based on discrimination and calibration in both cohorts. Discrimination was assessed using the concordance index and area under the receiver operating characteristic curve (AUC), which reflects the accuracy of the model. A 95% confidence interval (CI) was calculated for each AUC. Calibration was performed by comparing the mean nomogram estimated survival and the mean observed survival. Statistical analysis was conducted using SPSS version 22.0 (Chicago, IL, USA) and R version 3.0.0.

## Results

A total of 1,192 patients were included, with 795 and 397 patients in the model development and validation cohorts, respectively. The basic characteristics of the patients in both cohorts are shown in **Table 1**. The 2018 FIGO stage IIIC1 and IIIC2 were observed in 178 patients (22.4%) and 55 patients (6.9%) in the model development cohort, and 101 patients (25.4%) and 24 patients (6.0%, *p* = 0.387) in the validation cohort, respectively. More patients in the model development cohort had no SCC than those in the validation cohort (12.5% vs. 7.6%, *p* = 0.010). Meanwhile, the other characteristics did not differ significantly.

**Table 1:** Patients’ characteristics in the model development and validation cohorts.

Characteristics	Model development cohort (*n* = 795)	Validation cohort (*n* = 397)	*p*-value
**Age**			
**Young (<65 years)**	701 (88.2%)	352 (88.7%)	0.804
**Elderly (≥65 years)**	94 (11.8%)	45 (11.3%)	
**Histology**			
**SCC**	696 (87.5%)	367 (92.4%)	0.010
**Non-SCC**	99 (12.5%)	30 (7.6%)	
**Tumor size**			
**<4 cm**	326 (41.0%)	158 (39.8%)	0.689
**≥4 cm**	469 (59.0%)	239 (60.2%)	
**2018 FIGO stage**			
**IB**	84 (10.6%)	41 (10.3%)	0.387
**IIA**	57 (7.2%)	20 (5.0%)	
**IIB**	340 (42.8%)	176 (44.3%)	
**IIIA**	21 (2.6%)	5 (1.3%)	
**IIIB**	53 (6.7%)	29 (7.3%)	
**IIIC1**	178 (22.4%)	101 (25.4%)	
**IIIC2**	55 (6.9%)	24 (6.0%)	
**IVA**	7 (0.9%)	1 (0.3%)	
**SCC antigen**			
**<8.6 ng/ml**	483 (65.8%)	234 (64.6%)	0.703
**≥8.6 ng/ml**	251 (34.2%)	128 (35.4%)	
**Bilateral pelvic LN**			
**Yes**	137 (17.2%)	71 (17.9%)	0.780
**No**	658 (82.8%)	326 (82.1%)	
**Pelvic LN number**			
**0–1**	642 (80.8%)	312 (78.6%)	0.378
**≥2**	153 (19.2%)	85 (21.4%)	
**Common iliac LN**			
**Yes**	57 (7.2%)	39 (9.8%)	0.113
**No**	738 (92.8%)	358 (90.2%)	
**Concurrent chemotherapy**			
**Yes**	676 (85.0%)	340 (85.6%)	0.779
**No**	119 (15.0%)	57 (14.4%)	

**Figure 1:**
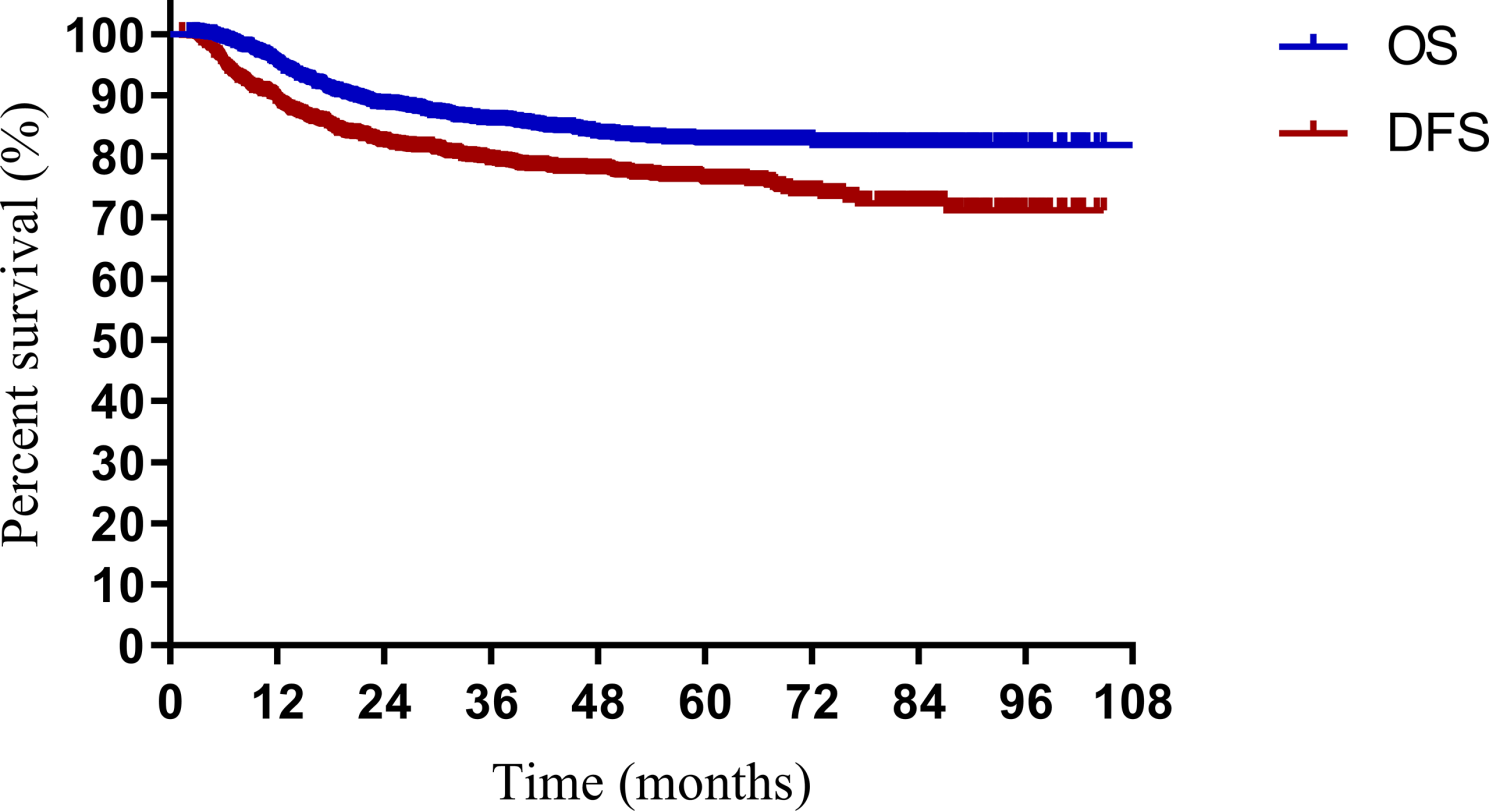
Overall survival (OS) and disease-free survival (DFS) for patients with cervical cancer being treated with concurrent chemoradiotherapy.

The median follow-up periods were 49.2 months (range, 2.9–108.5 months) and 48.5 months (range, 2.2–104.0 months) in the model development and validation cohorts, respectively. In the model development cohort, local failure, distant failure, and failure in both were observed in 69 (8.7%), 106 (13.3%), and 23 (2.9%) patients, respectively. In the validation cohort, local failure, distant failure, and failure in both were observed in 47 (11.8%), 44 (11.0%), and 16 (4.0%) patients, respectively. For all the patients, the 3- and 5-year survival rates were 85.5% and 82.3% for OS, and 76.1% and 73.1% for DFS, respectively (**Figure 1**). In both cohorts, the 3- and 5-year OS rates were 86.0% and 84.6% and 82.6% and 81.5% (*p* = 0.606), respectively. In both cohorts, the 3- and 5-year DFS rates were 76.5% and 75.1%, and 73.8% and 71.5% (*p* = 0.409), respectively.

**Figure 2:**
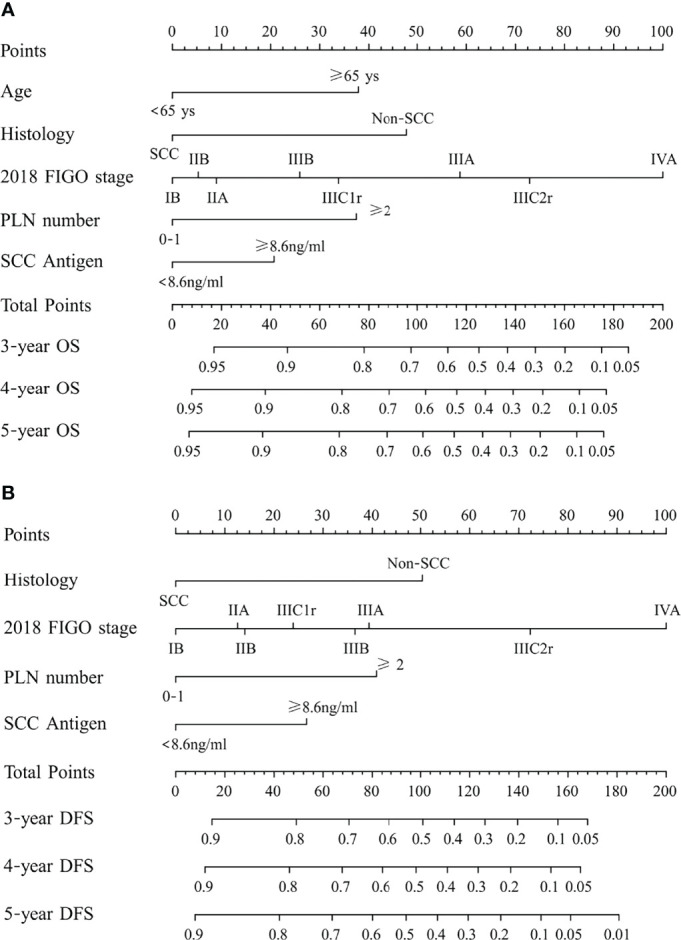
**(A)** Nomogram predicting overall survival (OS) and **(B)** disease-free survival (DFS) for patients with cervical cancer being treated with concurrent chemoradiotherapy. To use the nomogram, locate an individual patient’s characteristic on the variable row and draw a line upward to the point row to determine the points received for each variable value. The total score was determined by adding up the individual parameter points. Locate the total score on the total points axis and draw a line downward to the survival axes to determine the 3- and 5-year survival probability. PLN, pelvic lymph node; SCC, squamous cell carcinoma; non-SCC, nonsquamous cell carcinoma.

**Figure 3:**
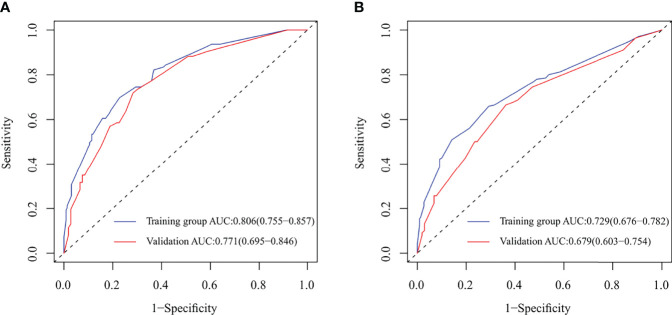
Receiver operating characteristic (ROC) curve and area under ROC curve (AUC) for nomograms predicting 5-year overall survival (OS) and disease-free survival (DFS) in the model development and validation cohorts. **(A)** ROC and AUC for nomogram predicting 5-year OS; **(B)** ROC and AUC for nomogram predicting 5-year DFS.

After univariate analysis, histology type, tumor size, 2018 FIGO stage, SCC antigen, pelvic lymph node number, and common iliac LNM were identified as predictors for OS and DFS, as shown in **Tables 2**, **3**. Age was a significant factor for OS. After multivariate analysis, age, histology type, 2018 FIGO stage IIIC2 and IVA, SCC antigen, and pelvic lymph node number were independent factors for OS. Histology type, 2018 FIGO IIIC2 and IVA, SCC antigen, and pelvic lymph node number were significant predictors of DFS. Nomograms predicting 3-, 4-, and 5-year OS and DFS were constructed based on these factors (**Figures 2**, **3**). As FIGO staging systems were associated with the survival of patients and the present study focused on the 2018 FIGO staging system, the 2018 FIGO stages IB, IIA, IIB, IIIA, and IIIB were also incorporated into the nomograms. However, these were not significant predictors of the OS and DFS in the multivariate analysis.

**Table 2:** Univariate and multivariate analyses for overall survival in the model development cohort.

Variables	Univariate analysis		Multivariate analysis	
	HR (95% CI)	*p*-value	HR (95% CI)	*p*-value
**Age**				
**Young (<65 years)**	Reference		Reference	
**Elderly (≥65 years)**	1.86 (1.16–2.99)	0.010	2.49 (1.42–4.35)	0.001
**Histology type**				
**SCC**	Reference		Reference	
**Non-SCC**	2.38 (1.56–3.62)	<0.001	3.16 (1.83–5.46)	<0.001
**Tumor size**				
**<4 cm**	Reference			
**≥4 cm**	2.49 (1.63–3.79)	<0.001		
**2018 FIGO stage**				
**IB**	Reference		Reference	
**IIA**	1.23 (0.33–4.58)	0.758	1.24 (0.33–4.62)	0.748
**IIB**	1.53 (0.59–3.92)	0.381	1.14 (0.43–3.01)	0.799
**IIIA**	4.81 (1.39–16.63)	0.013	4.09 (1.07–15.59)	0.039
**IIIB**	2.87 (0.94–8.77)	0.065	1.87 (0.53–6.61)	0.334
**IIIC1**	3.99 (1.57–10.15)	0.004	2.26 (0.78–6.56)	0.135
**IIIC2**	13.30 (5.13–34.48)	<0.001	5.77 (1.89–17.67)	0.002
**IVA**	10.92 (2.61–45.75)	0.001	11.06 (2.51–48.83)	0.002
**SCC antigen**				
**<8.6 ng/ml**	Reference		Reference	
**≥8.6 ng/ml**	1.98 (1.34–2.91)	0.001	1.65 (1.05–2.58)	0.030
**Pelvic lymph node number**				
**0–1**	Reference		Reference	
**≥2**	2.09 (1.75–2.50)	<0.001	1.57 (1.15–2.15)	0.005
**Common iliac lymph node metastasis**				
**No**	Reference			
**Yes**	5.58 (3.65–8.53)	<0.001		

**Table 3:** Univariate and multivariate analyses for disease-free survival in the model development cohort.

Variables	Univariate analysis		Multivariate analysis	
	HR (95% CI)	*p*-value	HR (95% CI)	*p*-value
**Age**				
**Young (<65 years)**	Reference			
**Elderly (≥65 years)**	1.29 (0.85–1.94)	0.235		
**Histology**				
**SCC**	Reference		Reference	
**Non-SCC**	2.29 (1.64–3.20)	<0.001	2.98 (1.95–4.54)	<0.001
**Tumor size**				
**<4 cm**	Reference			
**≥4 cm**	2.08 (1.52–2.84)	<0.001		
**2018 FIGO stage**				
**IB**	Reference		Reference	
**IIA**	1.37 (0.53–3.55)	0.518	1.31 (0.49–3.53)	0.589
**IIB**	1.69 (0.84–3.40)	0.142	1.36 (0.67–2.78)	0.398
**IIIA**	3.15 (1.12–8.86)	0.029	2.36 (0.78–7.13)	0.128
**IIIB**	2.71 (1.16–6.34)	0.021	2.21 (0.90–5.43)	0.084
**IIIC1**	3.71 (1.84–7.48)	<0.001	1.69 (0.75–3.82)	0.209
**IIIC2**	10.87 (5.25–22.52)	<0.001	4.81 (2.02–11.47)	<0.001
**IVA**	12.48 (4.18–37.30)	<0.001	8.47 (2.52–28.52)	0.001
**SCC antigen**				
**<8.6 ng/ml**	Reference		Reference	
**≥8.6 ng/ml**	1.92 (1.42–2.58)	<0.001	1.79 (1.27–2.51)	0.001
**Pelvic lymph node number**				
**0–1**	Reference			
**≥2**	1.95 (1.69–2.25)	<0.001	1.56 (1.21–2.02)	0.001
**Common iliac LNM**				
**No**	Reference			
**Yes**	4.59 (3.21–6.58)	<0.001		

**Figure 4:**
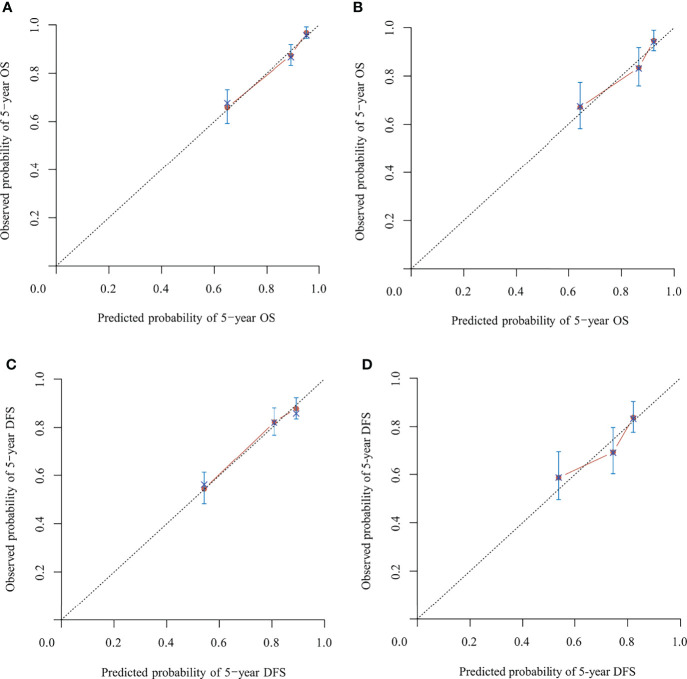
Calibration of the nomograms predicting 5-year overall survival (OS) and disease-free survival (DFS) in the model development and validation cohorts, respectively. **(A)** Five-year OS in the model development cohort. **(B)** Five-year OS in the validation cohort. **(C)** Five-year DFS in the model development cohort. **(D)** Five-year the DFS in validation cohort. The dashed line represents the ideal nomogram, and the solid line represents the observed nomogram. The predicted probability line was close to the ideal line.

In the model development and validation cohorts, the concordance index values were 0.78 and 0.75 for the nomogram predicting OS and 0.74 and 0.67 for the nomogram predicting OS and DFS, respectively. In the model development and validation cohorts, the AUCs were 0.81 and 0.77 for the nomogram predicting 5-year OS (**Figure 3A**) and 0.73 and 0.68 for the nomogram predicting 5-year DFS (**Figure 3B**), respectively. The receiver operating characteristic (ROC) curve of nomograms predicting 3-year OS and DFS in the model development and validation cohorts are shown in **Supplementary Figure S1**. The calibration of the nomograms predicting 3- and 5-year OS and DFS in both cohorts are shown in **Figure 4** and **Supplementary Figure S2**. The dashed line represents the ideal nomogram, and the solid line represents the observed nomogram. The predicted probability line was close to the ideal line. This suggests a good agreement between the predictions and actual values.

## Discussion

Previously, we constructed a nomogram predicting the OS, DFS, local control, and distant metastasis-free rates in patients with cervical cancer based on the 2009 FIGO staging system (11). In that study, we focused on regional LNM. Lymph nodes were classified by region (paraaortic and pelvic), number, diameter, and laterality (bilateral/ipsilateral). After univariate and multivariate analyses, paraaortic LNM, pelvic LNM, and pelvic lymph node number were incorporated into our nomograms for predicting OS and DFS (11). Currently, paraaortic LNM (stage IIIC2) and pelvic LNM (stage IIIC1) have been incorporated into the 2018 FIGO staging system.

Currently, nomograms based on the 2018 FIGO staging system are limited. In 2021, Tang et al. conducted a large multicenter study of 3,238 patients, of whom 2,009 cervical cancer patients had FIGO stages IA1–IIA2 disease and had undergone surgeries. Patients were restaged according to the 2018 FIGO staging system. Subsequently, nomograms were constructed based on the 2018 FIGO stage, histology, and parametrial involvement (14). Zang et al. constructed a nomogram predicting survival for squamous cell cervical cancer patients with 2018 FIGO II and III. However, in this study, the 2018 FIGO stage was not analyzed in univariate and multivariate analyses and was not used in the nomogram (15). For patients with locally advanced cervical cancer patients treated with concurrent chemoradiotherapy, most nomograms developed after 2019 still use the 2009 or 2014 FIGO staging system (16–18). To the best of our knowledge, this is the first nomogram based on the 2018 FIGO staging system for patients with locally advanced cervical cancer treated with concurrent chemoradiotherapy.

In the 2018 FIGO staging system, the survival of patients with IIIC1 varies depending on the local tumor factors (19). Patients with stage IIIC1 disease may have much better survival than patients with stages IIIA and IIIB (19, 20). A study by the National Cancer Database included 62,212 patients with cervical cancer. For patients with stages IIIA, IIIB, and IIIC1 diseases, the 5-year survival rates were 40.7%, 41.4%, and 60.8%, respectively (20). In another study based on the SEER database, the 5-year cause-specific survival of patients with stages IIIA, IIIB, and IIIC1 cervical cancer were 46.0%, 42.6%, and 62.1%, respectively (19). Similarly, in this study, stage IIIC1 cervical cancer had lower points than stages IIIA and IIIB cervical cancer in the nomogram predicting DFS and OS. This suggests that patients with stage IIIC1 cervical cancer benefit from further classification. According to the American Joint Committee on Cancer, the N stage is also classified using factors such as number (breast cancer, rectal cancer), bilateral/ipsilateral (nasopharyngeal carcinoma, other head and neck cancers), and diameter (head and neck cancers, cancer of the vulva) of lymph nodes in the staging system for some tumors. In the present study, the number of metastatic pelvic lymph nodes became significant in predicting OS and DFS once the 2018 FIGO staging was used in the multivariate analysis. The points for ≥2 pelvic lymph nodes in nomograms were comparatively high. A patient with two or more pelvic lymph nodes will score 65 points in the nomogram predicting DFS with 24 points from IIIC1 and 41 points from thepelvic number for pelvic LNM ≥2. It is only 9 points lower than that of IIIC2 (72 points). If the FIGO staging system undergoes further subclassification in the future, the number of pelvic lymph nodes has the potential to be adopted.

In this study, histology and SCC antigen were also considered in the nomogram predicting OS and DFS. Age was considered only for predicting OS. It is clear that patients with adenocarcinoma, adenosquamous carcinoma, or neuroendocrine carcinoma have poorer survival than patients with SCC (21–24). Excessive pretreatment with SCC antigen is associated with poor survival (13, 25). These factors were added to the nomograms to improve their accuracy.

This study had some limitations. Both cohorts were from the same database of a single institute. External validation with an independent cohort was not conducted. Hence, the nomograms should be externally validated with an independent cohort before clinical use. Tumor-differentiation grade and lymph-vascular space invasion (LVSI) are important prognostic factors for patients with cervical cancer (26–31). However, data on tumor-differentiation grade and LVSI were largely absent in our database and were not analyzed in the present study. Although the reason for this was to avoid inroducing bias by skewing the data, it may lead to bias on omission. Additionally, the median follow-up period was just 49.2 months, which is not long enough. Furthermore, as histology was not balanced between the model development and validation cohorts, this may influence on the results of external validation.

In summary, we have constructed nomograms to predict the OS and DFS of patients with cervical cancer being treated with definitive concurrent chemoradiotherapy or radiotherapy using the 2018 FIGO staging system.

## Data Availability Statement

The raw data supporting the conclusions of this article will be made available by the authors, without undue reservation.

## Ethics Statement

The studies involving human participants were reviewed and approved by institutional review board of Peking Union Medical College Hospital. Written informed consent for participation was not required for this study in accordance with the national legislation and the institutional requirements.

## Author Contributions

FZ contributed to the conception of the study. QM and WW wrote the first draft of the manuscript. QM, WW, XL, and DW contributed to data collection. All authors contributed to the article and approved the submitted version.

## Funding

This work was supported by the National Key Technologies Research and Development Program of China (grant number 2016YFC0105207).

## Conflict of Interest

The authors declare that the research was conducted in the absence of any commercial or financial relationships that could be construed as a potential conflict of interest.

## Publisher’s Note

All claims expressed in this article are solely those of the authors and do not necessarily represent those of their affiliated organizations, or those of the publisher, the editors and the reviewers. Any product that may be evaluated in this article, or claim that may be made by its manufacturer, is not guaranteed or endorsed by the publisher.

## Supplementary Material

Supplementary Figure 1 Receiver operating characteristic (ROC) curve and area under ROC curve (AUC) for nomograms predicting 3-year overall survival (OS) and disease-free survival (DFS) in model development and validation cohorts. **(A)** ROC and AUC for nomogram predicting 3-year OS; **(B)** ROC and AUC for nomogram predicting 3-year DFS.

Supplementary Figure 2 Calibration of the nomograms predicting 3-year overall survival (OS) and disease-free survival (DFS) in the model development and validation cohorts. **(A)** Three-year OS in the model development cohort; **(B)** Three-year OS in the validation cohort; **(C)** Three-year DFS in the model development cohort; **(D)** Three-year DFS in the validation cohort.

## Abbreviations

LNM, lymph node metastasis; FIGO, International Federation of Gynecology and Obstetrics; OS, overall survival; DFS, disease-free survival; CTV, clinical target volume.
