# Supplementary figures and images for: Nomograms Predicting Survival of Cervical Cancer Patients Treated With Concurrent Chemoradiotherapy Based on the 2018 FIGO Staging System
Source: Front Oncol. 2022 May 11;12:870670. doi: 10.3389/fonc.2022.870670 (PMC9130963; doi:10.3389/fonc.2022.870670)

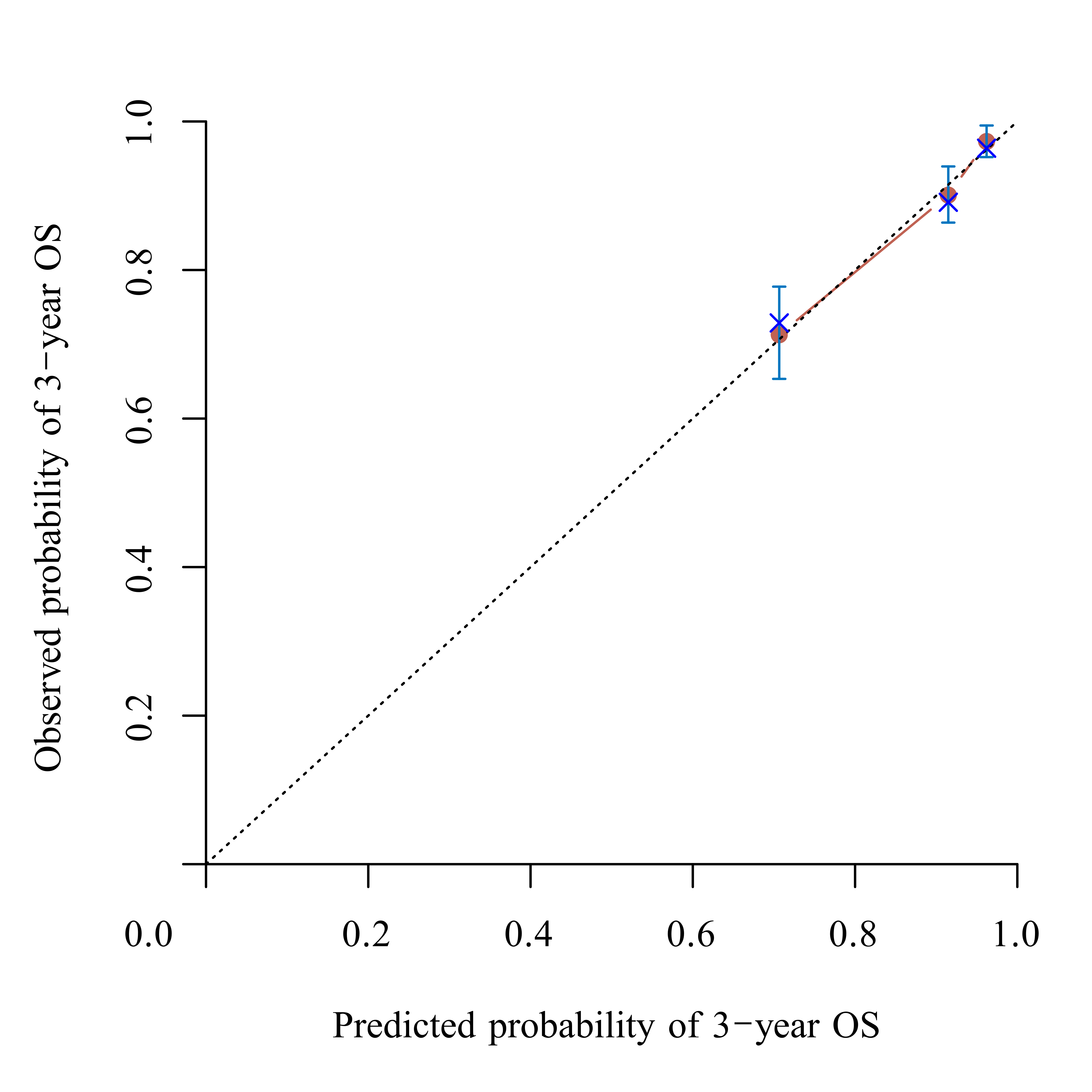

Supplement: Supplementary file 3 [file Image_3.tif]

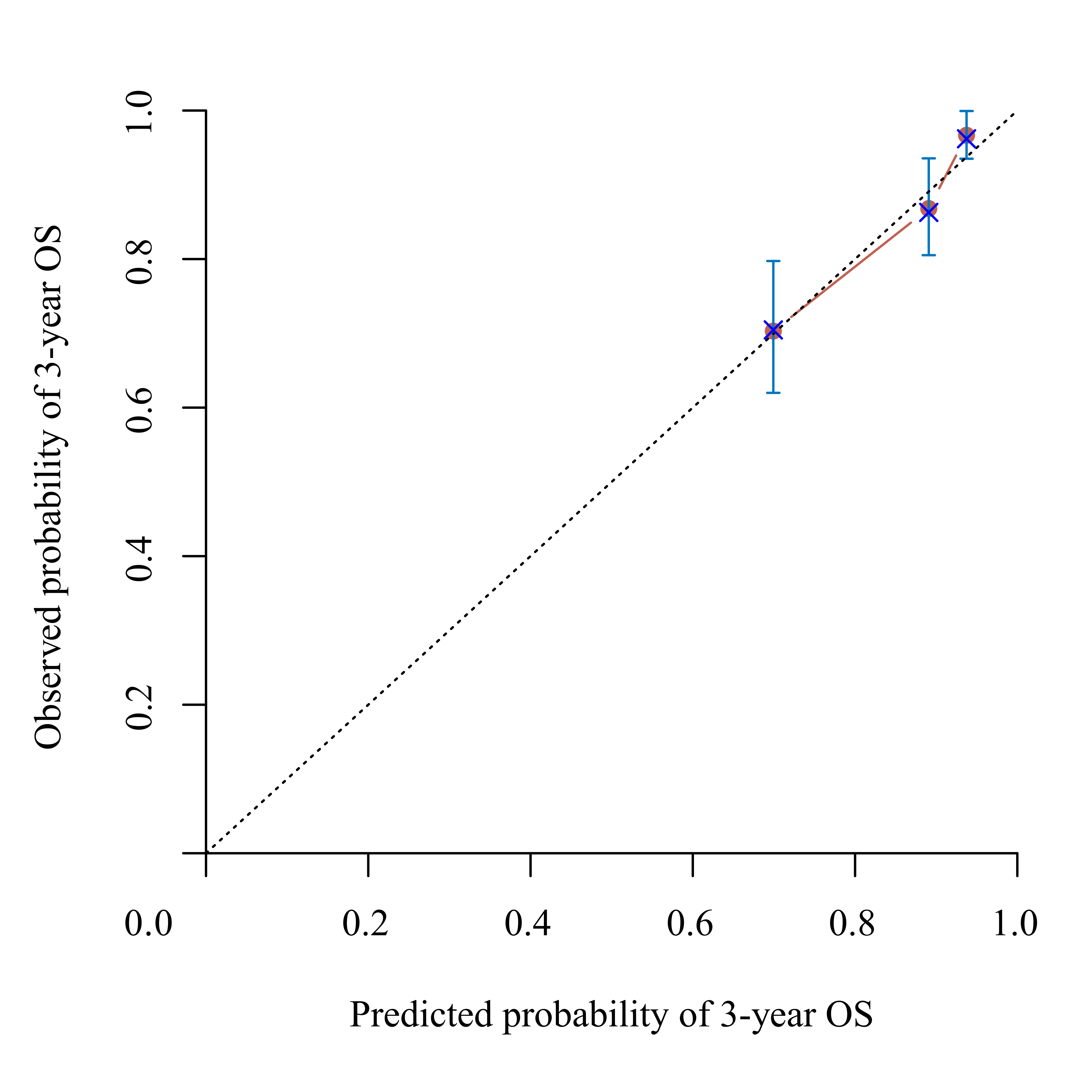

Supplement: Supplementary file 4 [file Image_4.tif]

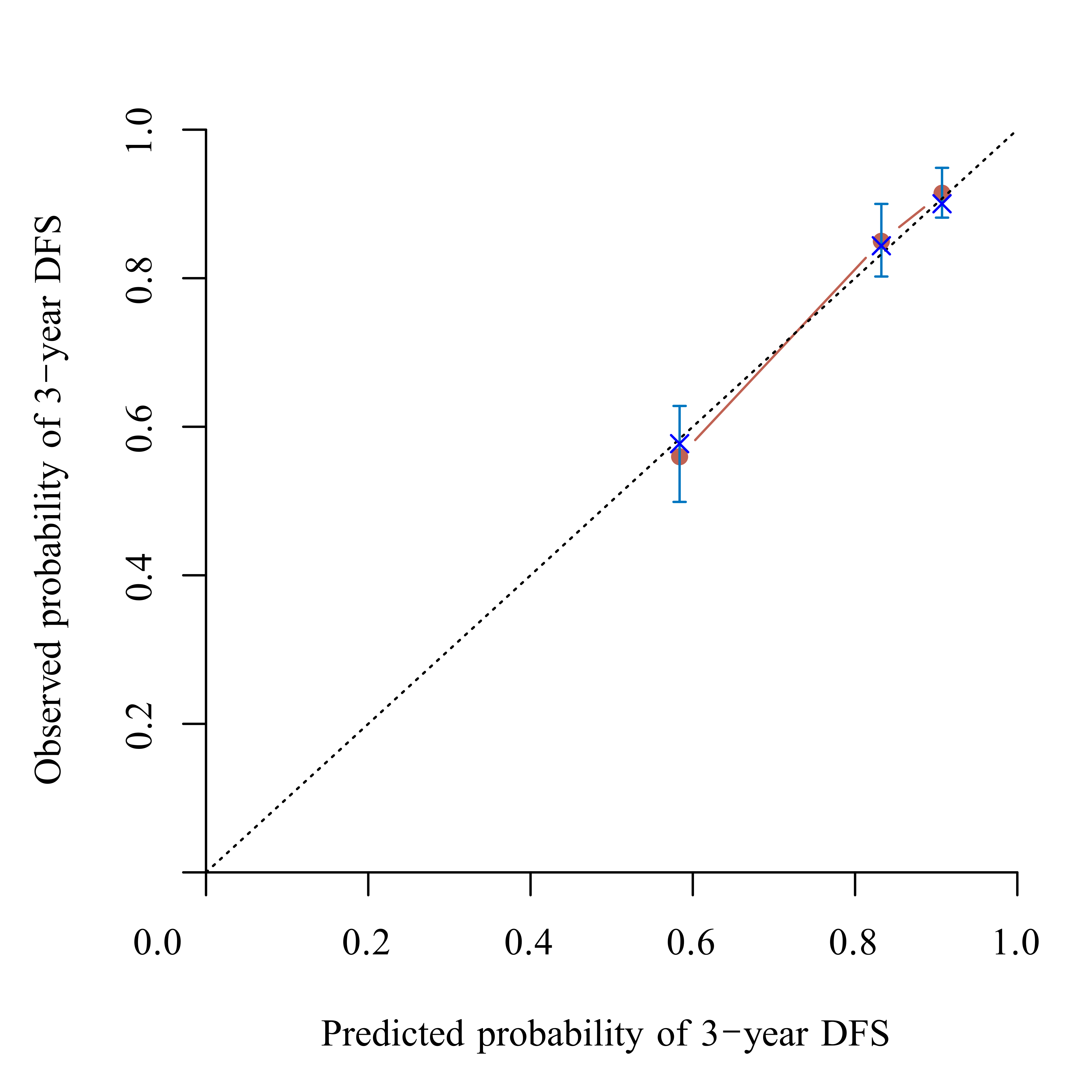

Supplement: Supplementary file 5 [file Image_5.tif]

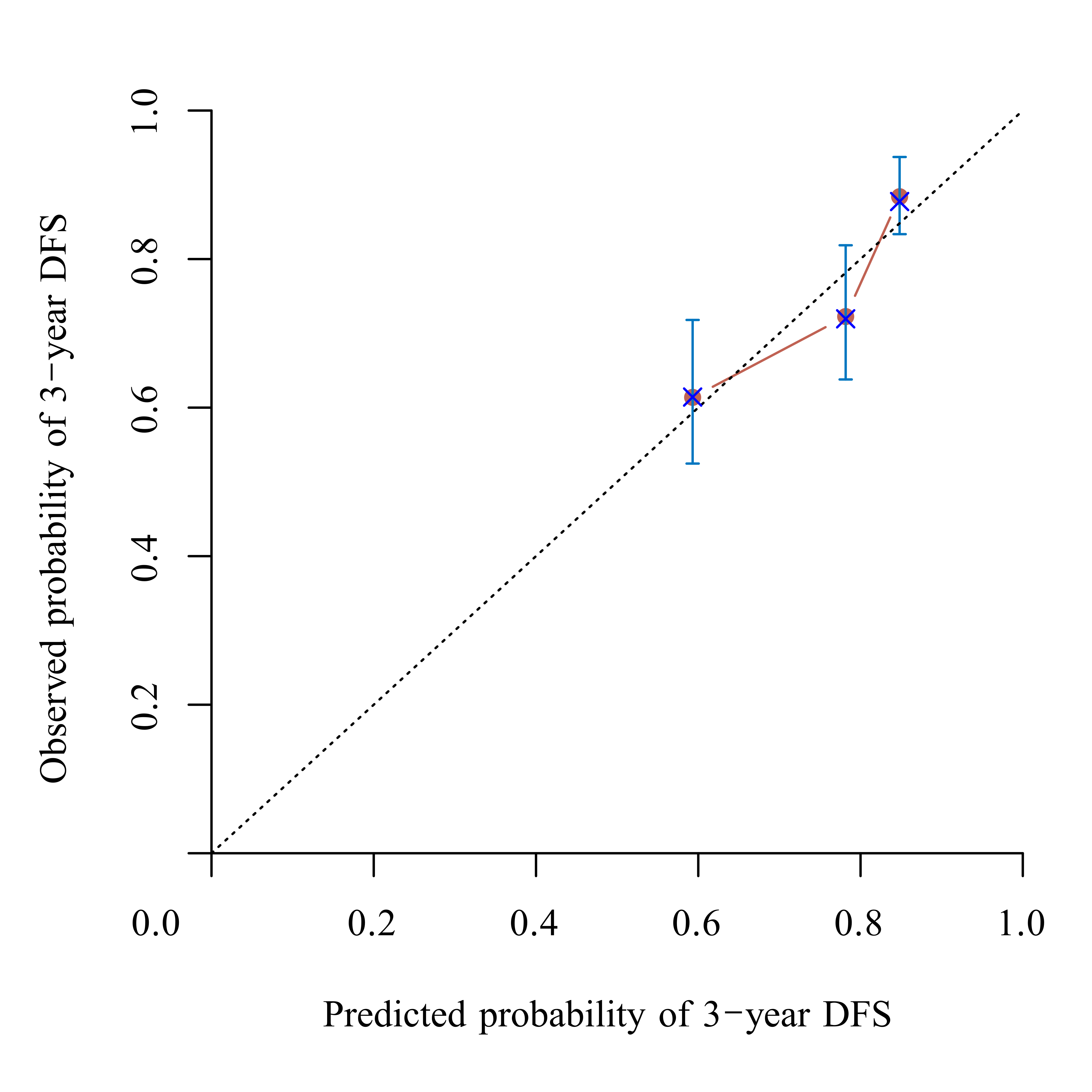

Supplement: Supplementary file 6 [file Image_6.tif]
